# Oseltamivir treatment of influenza A and B infections in infants

**DOI:** 10.1111/irv.12862

**Published:** 2021-05-03

**Authors:** Janna‐Maija Mattila, Tytti Vuorinen, Matti Waris, Petri Antikainen, Terho Heikkinen

**Affiliations:** ^1^ Department of Pediatrics University of Turku Turku University Hospital Turku Finland; ^2^ Department of Clinical Microbiology Institute of Biomedicine Turku University Hospital University of Turku Turku Finland; ^3^ ArcDia International Ltd Turku Finland

**Keywords:** infant, influenza A, influenza B, oseltamivir, viral load

## Abstract

**Background:**

Oseltamivir treatment is currently the only way of managing influenza in young infants for whom influenza vaccines are not licensed, but little data exist on the effectiveness of the treatment in this age group.

**Methods:**

In a prospective study, we enrolled 431 newborn infants and followed them up for 10 months during their first respiratory season (September 2017‐June 2018). During each respiratory illness, we examined the infants and obtained nasopharyngeal specimens for determination of the viral etiology. Infants with influenza were re‐examined at short intervals, and additional nasopharyngeal specimens were obtained at each visit for measuring the viral load. All infants with symptoms <48 hours received oseltamivir treatment. The parents filled out daily symptom diaries.

**Results:**

Among 23 infants with influenza A, the mean total duration of illness in oseltamivir recipients was 82.1 hours, compared with 253.5 hours in infants without treatment (*P* = .0003). For infants with influenza B, the corresponding durations were 110.0 and 173.9 hours, respectively (*P* = .03). In infants with influenza A, total symptom scores were significantly lower in oseltamivir‐treated infants at all time points between days 3 and 11 after the onset of therapy. In most children with either influenza A or B, viral antigen concentrations declined rapidly within 1‐2 days after the initiation of oseltamivir treatment.

**Conclusions:**

Oseltamivir treatment of infants with influenza rapidly decreased the viral load in nasopharyngeal secretions and shortened the duration and severity of symptoms. The clinical effectiveness of oseltamivir appeared to be greater against influenza A than against influenza B infections.

## 1 INTRODUCTION

Among all children, the burden of influenza is greatest on the youngest ones. The rates of influenza‐associated deaths and hospitalizations are highest among infants during the first 6 months of life, and substantial numbers of infants in this age group present with severe sepsis‐like illnesses.^1^, ^2^, ^3^, ^4^, ^5^, ^6^ However, even in the youngest age groups, most infants with influenza are managed as outpatients.^7^, ^8^, ^9^

Prevention of influenza in infants is challenging because influenza vaccines are not licensed for use in children younger than 6 months of age, and even in older infants, the immune response to influenza vaccination may be suboptimal compared with that later in childhood.^10^ Although maternal influenza vaccination during pregnancy provides some protection against influenza for young infants, the duration of protection is limited to the first few months of life.^11^ In the absence of vaccine‐based prevention of influenza in young infants, the only available way of reducing the clinical impact of influenza is by the use of oseltamivir treatment. Oseltamivir is the only antiviral drug licensed and recommended for use also in young infants.^12^ Oseltamivir treatment should be started within 2 days after getting sick, but its efficacy is highest when started as soon as possible after the onset of symptoms.^13^, ^14^, ^15^

Despite the clinical importance of oseltamivir treatment in young infants, there are scarce data on the effectiveness of this drug especially among infants treated as outpatients.^16^, ^17^ The aim of this study was to evaluate the effectiveness of oseltamivir in reducing the duration of illness, severity of clinical symptoms, and viral load in outpatient infants younger than 1 year of age with influenza A or B infection.

## 2 METHODS

### 2.1 Subjects and study design

This prospective cohort study was carried out at a primary care study clinic in Turku, Finland, as described earlier.^9^ Briefly, 431 infants born at Turku University Hospital in June‐August 2017 were enrolled in a cohort that was followed for 10 months from September 1, 2017, through June 30, 2018. Infants were eligible for participation if they lived within the catchment area of the hospital, the parents were able to communicate in Finnish language, and the infant did not have any major congenital defects or serious illnesses. Approximately half of all infants born during the enrollment period were enrolled in the follow‐up cohort. The Ethics Committee of the Hospital District of Southwest Finland approved the study protocol, and written informed consent was obtained from the parents of all participating infants. Before commencement of the study, the parents provided background information about the family, pregnancy, and delivery via a questionnaire.

### 2.2 Study procedures

The parents were asked to bring their child for clinical examination at the study clinic as soon as possible after the onset of fever or any signs of respiratory infection. The study clinic was open every day, and all visits were free of charge to the families. At each visit, a study physician examined the child, recorded the signs and symptoms, clinical findings, and treatment in a structured medical record, and obtained nasopharyngeal specimens for determination of the viral etiology of the illness. To enable diagnosing any complications that might develop later during the course of the illness, the infants were re‐examined whenever the parents deemed it necessary, and in any case on scheduled visits 5‐7 days after illness onset. Infants diagnosed with influenza were routinely re‐examined at shorter intervals, mostly at intervals of 2 days.

### 2.3 Virologic methods

At the initial visit for each respiratory illness, regardless of the severity of symptoms or the presence or absence of fever, two nasopharyngeal flocked swab specimens (Ultra minitip, Copan Italia S.p.a, Italy) were collected for viral analyses. One of the specimens was analyzed by multiplex reverse transcription‐polymerase chain reaction (RT‐PCR) assays for 16 viruses at the Department of Clinical Microbiology, Turku University Hospital (Allplex^™^ Respiratory Panels 1‐3, Seegene Inc.). The other specimen was analyzed immediately at the study clinic by study nurses using an automated rapid antigen test that identified 11 respiratory pathogens and provided initial results within 20 minutes (mariPOC Respi test, ArcDia International Ltd.). The antigen test differentiated between influenza A and B viruses, and it also provided semi‐quantitative data on antigen concentrations in the specimens. Compared with PCR, the reported sensitivities of the antigen test for influenza A and B viruses are 92.3% and 87.5%, with corresponding specificities of 99.8% and 100%, respectively.^18^ To allow for evaluation of viral loads during the course of the illness, a specimen for antigen testing was obtained during each re‐examination of an infant with confirmed influenza infection until antigen detection turned negative. Calculation of antigen concentrations in the specimens was based on dose‐response curves of standardized influenza A and B virus nucleoprotein antigen preparations.

### 2.4 Oseltamivir treatment

All infants who were diagnosed with influenza A or B infection within 48 hours of illness onset received oral oseltamivir treatment. The dosage of oseltamivir was 3 mg/kg twice daily for 5 days. The first dose of oseltamivir was given already at the study clinic to all infants in whom the initial antigen test result was positive for influenza.

### 2.5 Symptom diaries

The parents of all infants were asked to complete daily symptom diaries throughout the 10‐month follow‐up period. For infants diagnosed with influenza, the parents were additionally asked to fill out a separate, more detailed influenza symptom diary twice daily (in the morning and in the evening) until their child was asymptomatic, but at least for 8 days. The day of the first dose of oseltamivir treatment (or the day of the first visit to the study clinic for infants who did not receive oseltamivir treatment) was recorded as day 1 in the influenza symptom diary. At each time point, the parents recorded the infant´s measured temperature, the presence and severity of cough, rhinitis, vomiting, and diarrhea (on a 4‐point scale), the administration of oseltamivir, and the doses of antipyretic/analgesic medications administered during the preceding 12 hours.^13^

### 2.6 Definitions

The total influenza symptom score at each time point was calculated by summing the scores for fever, cough, rhinitis and antipyretic/analgesic medications (Table 1). Total symptom scores were analyzed starting from the evening of day 1.

**TABLE 1:** Scoring of symptoms in infants with influenza

Variable	Degree of severity	Score
Temperature (^o^C)	<37.5	0
	37.5‐37.9	1
	38.0‐38.4	2
	38.5‐38.9	3
	39.0‐39.4	4
	39.5‐39.9	5
	≥40.0	6
Cough and rhinitis (both symptoms scored separately)	Absent	0
	Mild	1
	Moderate	2
	Severe	3
Antipyretic/analgesic medications administered during past 12 h	Each dose of medication	1

The duration of preceding symptoms was calculated as the time interval from the onset of illness symptoms reported by the parents to the time of the initial visit at the study clinic. The total duration of illness was defined as the time interval from the onset of illness symptoms to the first time when the following conditions were met simultaneously and lasted so for ≥24 hours: temperature <37.5⁰C, and rhinitis and cough either absent or mild.

### 2.7 Statistical analyses

Differences in means were compared by the unpaired *t* test, and medians were compared by the Mann‐Whitney *U* test. Two‐sided *P* values of <.05 were considered to indicate statistical significance. All statistical analyses were performed using StatsDirect software, version 3.2.7 (StatsDirect Ltd.).

## 3 RESULTS

### 3.1 Patients and influenza illnesses

Of a total of 55 episodes of laboratory‐confirmed influenza diagnosed in the follow‐up cohort, 5 were excluded from these analyses because of double viral infections in which influenza virus was not the predominant virus (3 cases with rhinovirus and 2 with respiratory syncytial virus). Among the remaining 50 infants, the influenza symptom diary was not available for 10 infants, and the duration of illness could not be reliably determined in 2 cases, leaving 38 infants in the final analyses. The mean age of these infants at the diagnosis of influenza was 7.6 months (range 4.3‐10.9), and 21 (55.3%) of them were boys. Influenza A was detected in 23 (60.5%) and influenza B in 15 (39.5%) infants; all these infections were positive for influenza by both antigen detection and RT‐PCR. Influenza was diagnosed within 48 hours of symptom onset in 31 (81.6%) infants, and all of them received oseltamivir treatment.

### 3.2 Duration of illness

Among 31 infants treated with oseltamivir, the mean duration of symptoms preceding the first visit to the study clinic was 15.9 (SD 9.5) hours for infants with influenza A and 19.1 (SD 12.4) hours for those with influenza B (*P* = .43) (Table 2). In infants with influenza A, the mean total duration of illness in oseltamivir recipients was shortened by 171.4 hours (82.1 vs 253.5 hours) compared with infants without oseltamivir treatment (*P* = .0003). For infants with influenza B, the corresponding reduction in the total duration of illness among infants receiving oseltamivir was 63.9 hours (110.0 vs 173.9 hours; *P* = .03). No significant difference in the mean total duration of illness was observed between infants with influenza A and B treated with oseltamivir (82.1 vs 110.0 hours; *P* = .20).

**TABLE 2:** Duration of symptoms in infants with influenza A and B infection

Duration of symptoms	Influenza A			Influenza B		
	Oseltamivir treatment (n = 21)	No treatment (n = 2)	*P* value	Oseltamivir treatment (n = 10)	No treatment (n = 5)	*P* value
Before the initial visit (h)						
Mean (SD)	15.9 (9.5)	84.4 (15.0)	ND	19.1 (12.4)	66.5 (36.6)	ND
Median (IQR)	14.0 (8.1‐22.1)	84.4	ND	14.0 (10.4‐29.6)	80.8 (32.5‐93.4)	ND
After the initial visit (h)						
Mean (SD)	66.2 (51.2)	169.1 (21.0)	ND	90.8 (59.2)	107.5 (43.7)	ND
Median (IQR)	46.8 (30.1‐97.6)	169.1	ND	96.5 (27.9‐138.1)	91.5 (82.9‐140.0)	ND
Total duration of illness (h)						
Mean (SD)	82.1 (54.1)	253.5 (36.1)	.0003	110.0 (55.9)	173.9 (20.1)	.03
Median (IQR)	59.0 (47.3‐110.0)	253.5	.02	108.5 (55.6‐156.8)	180.0 (152.8‐192.0)	.04

Note

SD, standard deviation; IQR, interquartile range; ND, not done.

### 3.3 Symptom scores

**FIGURE 1:**
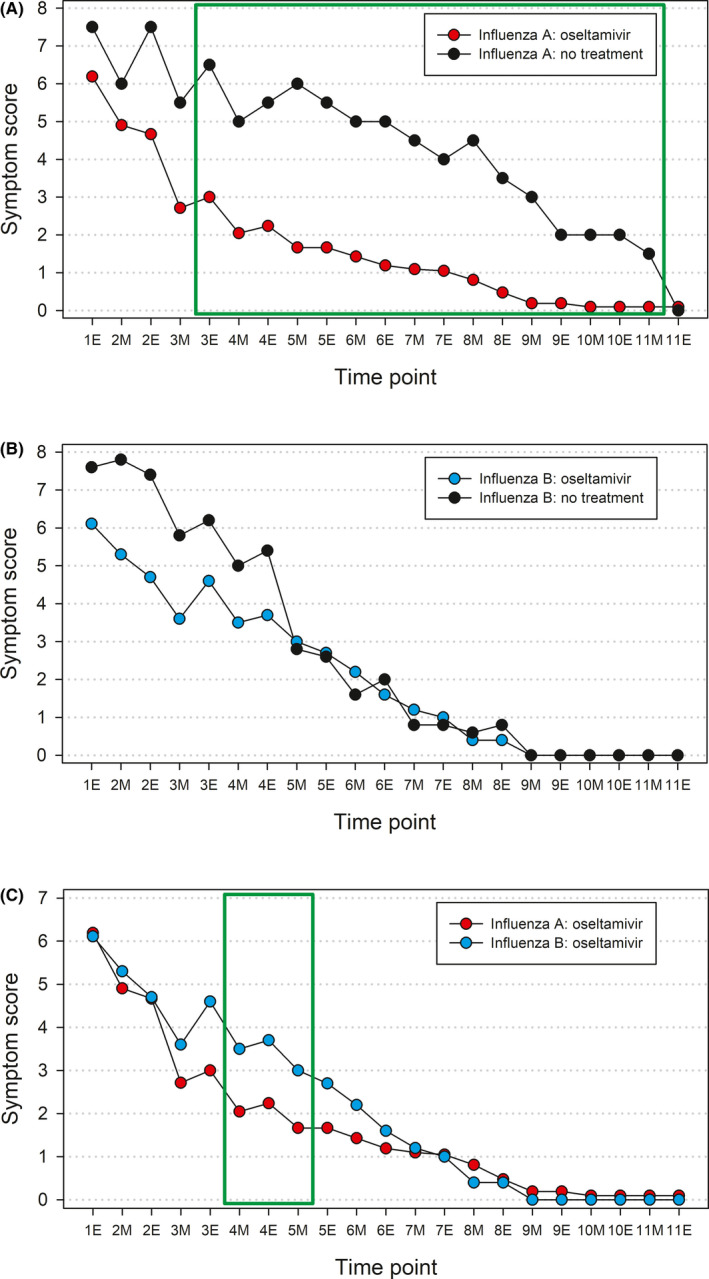
Total influenza symptom scores at different time points in infants with or without oseltamivir treatment. 1E denotes the evening of day 1, and 2M denotes the morning of day 2, etc. Day 1 is the day of the first dose of oseltamivir. A, Infants with influenza A; B, Infants with influenza B; C, oseltamivir‐treated infants with influenza A or B. Green boxes indicate the time points at which the differences between the groups were statistically significant

The total influenza symptom scores at different time points in infants with and without oseltamivir treatment are presented in Figure 1. In infants with influenza A, the mean symptom scores were significantly lower in oseltamivir‐treated infants at all time points from the evening of day 3 through the morning of day 11. For influenza B, the differences in mean symptom scores in infants with and without oseltamivir treatment did not reach statistical significance at any time point. When comparing oseltamivir‐treated infants with influenza A and B, the symptom scores were significantly lower in infants with influenza A from the morning of day 4 through the morning of day 5.

### 3.4 Viral load

**FIGURE 2:**
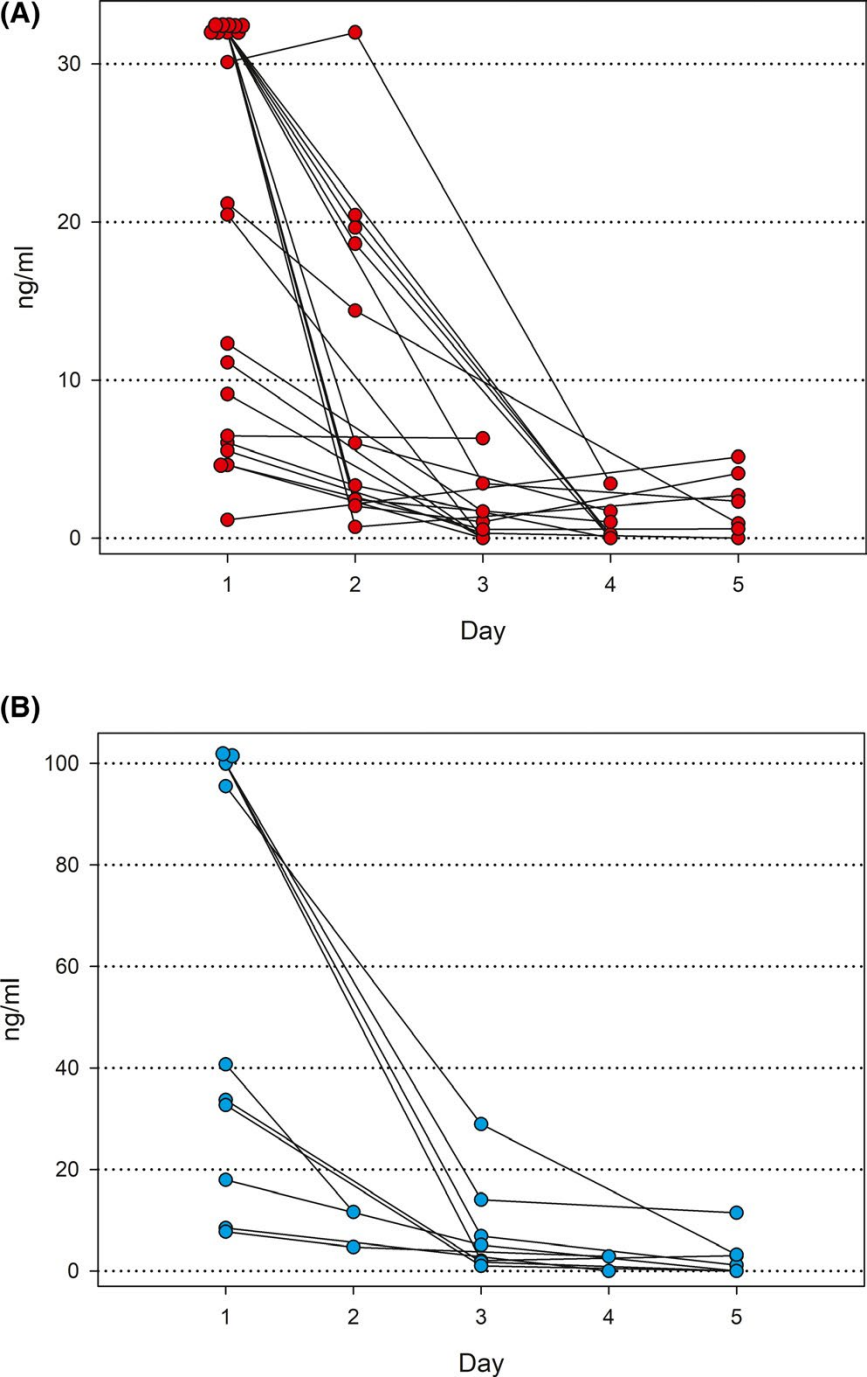
Nucleoprotein concentrations of influenza A and B viruses in nasopharyngeal specimens of infants treated with oseltamivir. Day 1 is the day of the first dose of oseltamivir. A, Influenza A (upper limit of semi‐quantitation 32 ng/mL). B, Influenza B (upper limit of semi‐quantitation 100 ng/mL). The initial specimens of 9 infants with influenza A and 3 infants with influenza B reached the upper limit of semi‐quantitation, indicating that the nucleoprotein concentrations were at the limit or higher

A mean number of 4.1 (range 2‐8) nasopharyngeal swab specimens were obtained from the infants for determination of the antigen concentrations of influenza viruses at various time points during the follow‐up. The antigen concentrations of influenza A and B viruses during the first 5 days of treatment are shown in Figure 2. In most children with either influenza A or B, viral antigen concentrations declined rapidly within 1‐2 days after the initiation of oseltamivir treatment.

### 3.5 Adverse events

Vomiting during oseltamivir treatment was reported by the parents in 15 (48.4%) of 31 infants. In 5 (33.3%) of these 15 infants, vomiting was already present before the first dose of oseltamivir. In 2 other infants, vomiting occurred before oseltamivir treatment but disappeared after the first dose of the drug. In most cases, vomiting was rated as mild and it ceased by the second day of treatment. The administration of oseltamivir was not discontinued in any infant because of vomiting. In infants without oseltamivir treatment, vomiting was reported in 2 (28.6%) of 7 cases.

Diarrhea during oseltamivir treatment was reported in 14 (45.2%) of 31 infants; in 4 (28.6%) of these 14 infants, diarrhea was present already before the first dose of oseltamivir. Among 7 infants not receiving oseltamivir, diarrhea was reported in 4 (57.1%) cases.

## 4 DISCUSSION

Our prospective study performed in a real‐life setting among outpatients provides new information about the benefits of oseltamivir treatment in young infants with influenza. Oseltamivir treatment initiated soon after illness onset shortened the duration and severity of symptoms and rapidly decreased the influenza viral load in nasopharyngeal secretions. The clinical importance of our findings derives from the fact that none of the currently available influenza vaccines is licensed for use in infants <6 months of age. As a result, antiviral treatment with oseltamivir is the only option for the management of influenza in young infants in whom the severity of this illness is greatest.

Our study was not a randomized controlled trial. In light of widespread recommendations for antiviral treatment of patients with influenza,^12^ it would have been ethically questionable to withhold effective therapy especially in this vulnerable group of young children. Because all infants diagnosed with influenza within 48 hours of symptom onset received oseltamivir, and those with a longer duration of symptoms did not receive the drug, it is obvious that the groups differed with respect to the duration of symptoms before the first visit to the study clinic. However, regarding any other variables, especially age and sex, the groups were fully comparable. Although the duration of symptoms before the first visit to the study clinic was different between oseltamivir‐treated and non‐treated infants, it did not affect the determination of the total duration of illness because the times of illness onset and the resolution of symptoms were known for each infant. It is also worth noting that although infants not treated with oseltamivir had a longer duration of symptoms before their first visit to the study clinic, the duration of symptoms also after their visit was longer than in those treated with oseltamivir. If the antiviral therapy had not had any impact on the course of the illness, infants with a longer duration of symptoms before the diagnosis of influenza should have been expected to recover sooner than those diagnosed with an early‐onset illness. Furthermore, our findings about the duration of illness after the start of oseltamivir treatment are well in line with the results from a previous randomized controlled trial in which oseltamivir treatment in young children was started within 24 hours of illness onset.^13^ In that study, the median time to resolution of illness after the administration of the first dose of oseltamivir was 3 days in children with influenza A.

The baseline symptom scores in infants not treated with oseltamivir appeared to be slightly higher than those in infants who received antiviral treatment, although the differences at baseline were not statistically significant. The mean duration of preceding symptoms before the initial visit to the study clinic was approximately 17 hours among infants who received oseltamivir and about 72 hours among those not receiving the treatment. As the symptoms of viral respiratory infections are generally considered to peak 2‐3 days after the onset of illness, it could be speculated that non‐treated infants were already at the peak of their symptoms during their initial visit, while symptoms in oseltamivir‐treated infants would have still increased after the first visit without the intervention with effective antiviral therapy. Of note, there were no differences in baseline symptom scores between oseltamivir‐treated infants with influenza A and B, among whom the durations of preceding symptoms were practically similar.

Some previous studies among children have concluded that the clinical effectiveness of oseltamivir is lower against influenza B than against influenza A,^13^, ^19^, ^20^ although some effectiveness has also been reported.^21^ The lower clinical effectiveness might be explained by reduced susceptibility of influenza B viruses to neuraminidase inhibitors that has been demonstrated in in vitro studies.^22^ In our study, the total duration of illness in infants with influenza B was significantly shorter among those who received oseltamivir compared with non‐treated infants, and also the reduction in influenza B viral load in oseltamivir recipients was rapid and comparable to that seen in infants with influenza A. However, due to relatively small numbers of children and the study design that was not optimal for direct comparison between infants with influenza A and B, the results should be interpreted with caution. Overall, our findings support the concept that oseltamivir has some clinical effectiveness against influenza B in children, but that effect appears to be smaller than that against influenza A.

The determination of changes in viral loads during the course of the illness was based on influenza A and B antigen concentrations in nasopharyngeal specimens obtained at the follow‐up visits. Although PCR is generally considered the most sensitive method for viral identification, PCR results may remain positive long after the period of true infectivity and the potential for influenza virus transmission. According to a recent study, antigen detection is far more informative than PCR for estimating the cessation of transmission of influenza viruses.^23^ As could be expected, short‐lived vomiting and diarrhea were reported in almost half of the infants treated with oseltamivir.^13^, ^16^ In many infants, however, these symptoms were present already before the first dosage of the drug. Moreover, substantial proportions of infants who did not receive oseltamivir had vomiting or diarrhea, indicating that these symptoms are frequent features of influenza illness in this age group.

It is well established that to gain maximal clinical effectiveness, the administration of oseltamivir should be started as soon as possible after the onset of illness.^13^, ^14^, ^15^ Because the accuracy of clinical diagnosis of influenza is notoriously poor in young children and several other viruses usually circulate in the community during influenza outbreaks,^24^, ^25^ confirmation of the influenza viral etiology of the illness is necessary for optimizing the use of oseltamivir in children. To enable appropriate use of antiviral therapy early in the course of the illness when the benefit from the treatment would be greatest, rapid influenza diagnostic assays with high sensitivity and specificity that can provide results already during the clinical visit would best streamline the treatment of influenza in outpatient settings.^26^

## CONFLICTS OF INTEREST

TH has received consulting fees from Roche. PA is an employee of ArcDia International Ltd. The other authors have no conflicts to report.

## AUTHOR CONTRIBUTION

**Janna‐Maija Mattila:** Conceptualization (supporting); Data curation (equal); Formal analysis (equal); Methodology (equal); Writing‐original draft (lead); Writing‐review & editing (supporting). **Tytti Vuorinen:** Methodology (equal); Writing‐review & editing (supporting). **Matti Waris:** Methodology (equal); Writing‐review & editing (supporting). **Petri Antikainen:** Methodology (equal); Writing‐review & editing (supporting). **Terho Heikkinen:** Conceptualization (lead); Data curation (equal); Formal analysis (equal); Funding acquisition (lead); Methodology (equal); Project administration (lead); Supervision (lead); Writing‐review & editing (lead).

### PEER REVIEW

The peer review history for this article is available at https://publons.com/publon/10.1111/irv.12862.

## Data Availability

The data that support the findings of this study are available on request from the corresponding author. The data are not publicly available due to privacy or ethical restrictions.

## References

[1] Wang X, Li Y, O´Brien KL, et al. Global burden of respiratory infections associated with seasonal influenza in children under 5 years in 2018: a systematic review and modelling study. Lancet Glob Health. 2020;8:e497‐e510. 32087815 10.1016/S2214-109X(19)30545-5 PMC7083228

[2] Poehling KA, Edwards KM, Weinberg GA, et al. The underrecognized burden of influenza in young children. N Engl J Med. 2006;355:31‐40. 16822994 10.1056/NEJMoa054869

[3] Silvennoinen H, Peltola V, Vainionpää R, Ruuskanen O, Heikkinen T. Incidence of influenza‐related hospitalizations in different age groups of children in Finland: a 16‐year study. Pediatr Infect Dis J. 2011;30:e24‐e28. 21298851 10.1097/inf.0b013e3181fe37c8

[4] Heikkinen T, Tsolia M, Finn A. Vaccination of healthy children against seasonal influenza: a European perspective. Pediatr Infect Dis J. 2013;32:881‐888. 23856713 10.1097/INF.0b013e3182918168

[5] Shang M, Blanton L, Brammer L, Olsen SJ, Fry AM. Influenza‐associated pediatric deaths in the United States, 2010–2016. Pediatrics. 2018;141:e20172918. 29440502 10.1542/peds.2017-2918

[6] Silvennoinen H, Peltola V, Vainionpää R, Ruuskanen O, Heikkinen T. Admission diagnoses of children 0–16 years of age hospitalized with influenza. Eur J Clin Microbiol Infect Dis. 2012;31:225‐231. 21643867 10.1007/s10096-011-1297-8

[7] Fell DB, Johnson J, Mor Z, et al. Incidence of laboratory‐confirmed influenza disease among infants under 6 months of age: a systematic review. BMJ Open. 2017;7:e016526. 10.1136/bmjopen-2017-016526 PMC5595206 28882916

[8] Teros‐Jaakkola T, Toivonen L, Schuez‐Havupalo L, et al. Influenza virus infections from 0 to 2 years of age: a birth cohort study. J Microbiol Immunol Infect. 2019;52:526‐533. 29254653 10.1016/j.jmii.2017.10.007

[9] Mattila JM, Thomas E, Lehtinen P, Vuorinen T, Waris M, Heikkinen T. Burden of influenza during the first year of life. Influenza Other Respir Viruses. 2020. 10.1111/irv.12820. (available online 18 Oct 2020) PMC8189221 33073478

[10] Walter EB, Rajagopal S, Zhu Y, Neuzil KM, Fairchok MP, Englund JA. Trivalent inactivated influenza vaccine (TIV) immunogenicity in children 6 through 23 months of age: do children of all ages respond equally? Vaccine. 2010;28:4376‐4383. 20447477 10.1016/j.vaccine.2010.04.058

[11] Omer SB, Clark DR, Madhi SA, et al. Efficacy, duration of protection, birth outcomes, and infant growth associated with influenza vaccination in pregnancy: a pooled analysis of three randomized controlled trials. Lancet Respir Med. 2020;8:597‐608. 32526188 10.1016/S2213-2600(19)30479-5 PMC7284303

[12] Uyeki TM, Bernstein HH, Bradley JS, et al. Clinical practice guidelines by the Infectious Diseases Society of America: 2018 update on diagnosis, treatment, chemoprophylaxis, and institutional outbreak management of seasonal influenza. Clin Infect Dis. 2019;68:895‐902. 30834445 10.1093/cid/ciy874 PMC6769232

[13] Heinonen S, Silvennoinen H, Lehtinen P, et al. Early oseltamivir treatment of influenza in children 1–3 years of age: a randomized controlled trial. Clin Infect Dis. 2010;51:887‐894. 20815736 10.1086/656408

[14] Malosh RE, Martin ET, Heikkinen T, Brooks WA, Whitley RJ, Monto AS. Efficacy and safety of oseltamivir in children: systematic review and individual patient data meta‐analysis of randomized controlled trials. Clin Infect Dis. 2018;66:1492‐1500. 29186364 10.1093/cid/cix1040

[15] Aoki FY, Macleod MD, Paggiaro P, et al. Early administration of oseltamivir increases the benefits of influenza treatment. J Antimicrob Chemother. 2003;51:123‐129. 12493796 10.1093/jac/dkg007

[16] Siedler K, Skopnik H. Oseltamivir for treatment of influenza in infants less than one year: a retrospective analysis. Pediatr Infect Dis J. 2010;29:495‐498. 20035245 10.1097/INF.0b013e3181cc4d01

[17] Shi L, Loveless M, Spagnuolo P, et al. Antiviral treatment of influenza in children: a retrospective cohort study. Adv Ther. 2014;31:735‐750. 25015536 10.1007/s12325-014-0136-6

[18] ArcDia International Ltd . Summary of mariPOC^®^ test performance. https://www.arcdia.com/maripoc/performance. Accessed February 16, 2021.

[19] Kawai N, Ikematsu H, Iwaki N, et al. A comparison of the effectiveness of oseltamivir for the treatment of influenza A and B: a Japanese multicenter study of the 2003–2004 and 2004–2005 influenza seasons. Clin Infect Dis. 2006;43:439‐444. 16838232 10.1086/505868

[20] Sugaya N, Mitamura K, Yamazaki M, et al. Lower clinical effectiveness of oseltamivir against influenza B contrasted with influenza A infection in children. Clin Infect Dis. 2007;44:197‐202. 17173216 10.1086/509925

[21] Dai Z, Zhang L, Yu Q, Liu L, Yang M, Fan K. Early administration of oseltamivir within 48 hours after onset of flulike symptoms can reduce the risk of influenza B virus‐associated pneumonia in hospitalized pediatric patients with influenza B virus infection. Pediatr Infect Dis J. 2020;39:e20‐e22. 31929434 10.1097/INF.0000000000002528

[22] Burnham AJ, Baranovich T, Govorkova EA. Neuraminidase inhibitors for influenza B virus infection: efficacy and resistance. Antiviral Res. 2013;100:520‐534. 24013000 10.1016/j.antiviral.2013.08.023 PMC3850058

[23] Inagaki K, Song MS, Crumpton JC, et al. Correlation between the interval of influenza virus infectivity and results of diagnostic assays in a ferret model. J Infect Dis. 2016;213:407‐410. 26068783 10.1093/infdis/jiv331 PMC4704662

[24] Zambon MC, Stockton JD, Clewley JP, Fleming DM. Contribution of influenza and respiratory syncytial virus to community cases of influenza‐like illness: an observational study. Lancet. 2001;358:1410‐1416. 11705487 10.1016/s0140-6736(01)06528-x

[25] Peltola V, Reunanen T, Ziegler T, Silvennoinen H, Heikkinen T. Accuracy of clinical diagnosis of influenza in outpatient children. Clin Infect Dis. 2005;41:1198‐1200. 16163640 10.1086/444508

[26] Williams LO, Kupka NJ, Schmaltz SP, Barrett S, Uyeki TM, Jernigan DB. Rapid influenza diagnostic test use and antiviral prescriptions in outpatient settings pre‐ and post‐2009 H1N1 pandemic. J Clin Virol. 2014;60:27‐33. 24630481 10.1016/j.jcv.2014.01.016

